# Impact of Dietary Advanced Glycation End Products on Female Reproduction: Review of Potential Mechanistic Pathways

**DOI:** 10.3390/nu14050966

**Published:** 2022-02-24

**Authors:** Marco Mouanness, Zaher Merhi

**Affiliations:** 1 Rejuvenating Fertility Center, 315 W 57th Street, Suite 208, New York, NY 10019, USA; marco.mouanness@rejuvenatingfertility.com; 2 Division of Reproductive Endocrinology and Infertility, Department of Obstetrics and Gynecology, Maimonides Medical Center, Brooklyn, NY 11219, USA; 3 Division of Reproductive Endocrinology and Infertility, Department of Obstetrics and Gynecology, SUNY Downstate Health Sciences University, Brooklyn, NY 11203, USA

**Keywords:** advanced glycation end product, infertility, PCOS, ovary, reproduction

## Abstract

Advanced glycation end products (AGEs), a heterogenous group of products formed by the reaction between protein and reducing sugars, can form endogenously due to non-enzymatic reactions or by exogenous sources such as diet where considerable increase in AGEs is observed due to the modification of food mainly by thermal processing. Recent studies have suggested that AGEs could impact, via inducing inflammation and oxidative stress, the reproductive health and fertility in both males and females. This review presents a summary of recently published data pertaining to the pathogenesis of dietary AGEs and their receptors as well as their potential impact on female reproductive health. More specifically, it will present data pertaining to dietary AGEs’ involvement in the mechanistic pathogenesis of polycystic ovary syndrome, ovarian dysfunction, as well as the AGEs’ effect perinatally on the female offspring reproduction. Understanding the mechanistic impact of dietary AGEs on female reproduction can help contribute to the development of targeted pharmacological therapies that will help curb rising female infertility.

## 1. Introduction

**Figure 1:**
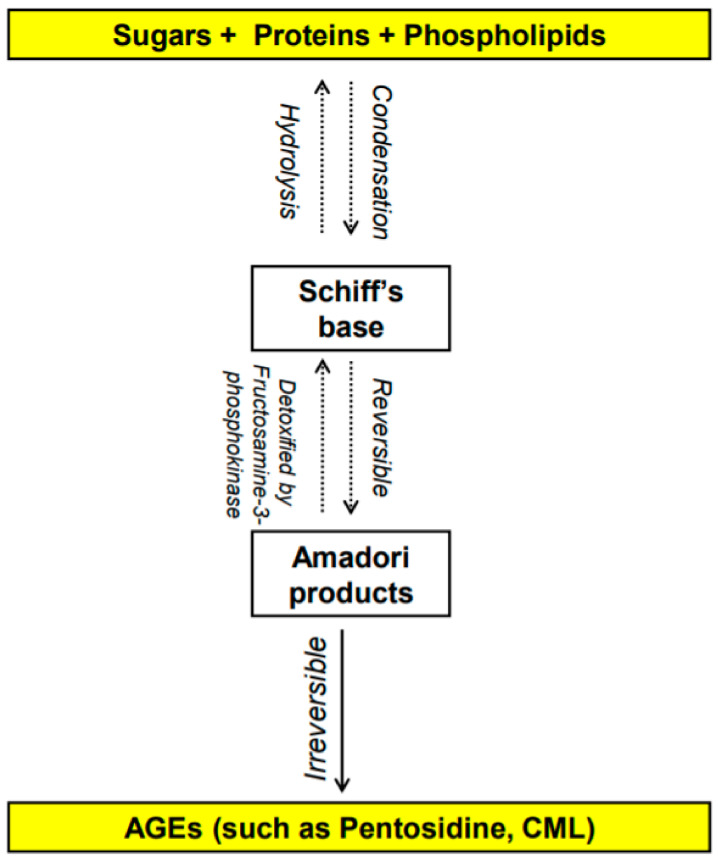
Overview of the formation of advanced glycation end products (AGEs) [1,2,3,4,5,6]. N-carboxymethyl-lysine (CML).

The Maillard reaction was first reported in 1912 by French scientist Louis Camille Maillard [1,2] and is defined as the chemical reaction in which the carbonyl group of carbohydrates reacts non-enzymatically with primary amino groups of proteins [3,4]. This reaction leads to the formation of advanced glycation end-products (AGEs). The early stages of the Maillard reaction lead to the formation of chemically reversible glycosylation products with proteins called Schiff bases and Amadori adducts [5]. The late stages of this glycation reaction forms complex glycation products which are the AGEs [6] (Figure 1). Since the 1980s, AGEs have been shown in several studies to be implicated in many health complications such as diabetes and aging [7], as well as many inflammatory diseases, obesity, cardiovascular diseases (CVD), metabolic syndrome and neurodegenerative disorders [8,9,10,11,12]. In the last decade, several studies have shown a potentially significant impact of AGEs on reproductive health in both males and females. This review will summarize the different types of AGEs and their receptors as well as the effect of dietary AGEs on female reproduction, in particular ovarian function, polycystic ovary syndrome (PCOS), and perinatally in utero on the female offspring reproduction. It also addresses the possible mechanistic pathways by which dietary AGEs alter female reproductive health.

### 1.1. What Are AGEs? How Do They Form?

AGEs are stable non-enzymatically catalyzed compounds which are formed by condensation of the amino groups of protein, lipid, amino acid and nucleic acid with the aldehyde group of reducing carbohydrate [13]. This nonenzymatic modification of proteins, lipids, and nucleic acids by glucose is one of the most important post-translational modifications in the formation of AGEs [14,15]. Once formed, the products of advanced glycation result in an irreversible cross-linking of proteins, loss of protein structure and function, followed by apoptosis and damage to cellular structures [14,15]. AGEs constitute a heterogeneous group of compounds of more than 20 members such as N-carboxymethyl-lysine (CML), pentosidine, 1,2-dicarbonyl precursor compounds glyoxal, and methylglyoxal. Pentosidine and CML are the most commonly studied AGEs [16,17] and have been used as markers of dietary AGE’s accumulation in various tissues [16,18,19,20,21,22]. Some of these compounds are fluorescent crosslinking (such as pentosidine [23]) products while others are non-fluorescent and/or non-crosslinking (such as CML [24,25]).

AGEs can be formed either endogenously by the body or from exogenous sources [1]. Endogenous AGEs are normally formed by glycosylation in different tissues of the body, occur slowly, increase progressively with aging and even faster with abnormal medical health conditions such as hyperglycemia and several chronic degenerative diseases [26]. Exogenous AGEs are obtained from food consumption and they are in very high levels in unhealthy food that is cooked at high temperature, such frying [27] and from smoking [28]. Contemporary methods of cooking (precooked fast-food meals), food high in protein and fat such as meat, cheese, and egg yolk dramatically increase serum AGEs’ concentration [27,29]. In addition to serum level, tissue AGEs can be influenced by diet as well [16,27]. Even though it is beyond the scope of the article, smoking has been identified as an exogenous source of AGEs [30]. Glycation products are present in tobacco and smoke in a form that can rapidly react with proteins to form AGEs [30].

### 1.2. How Do Dietary AGEs Act?

**Figure 2:**
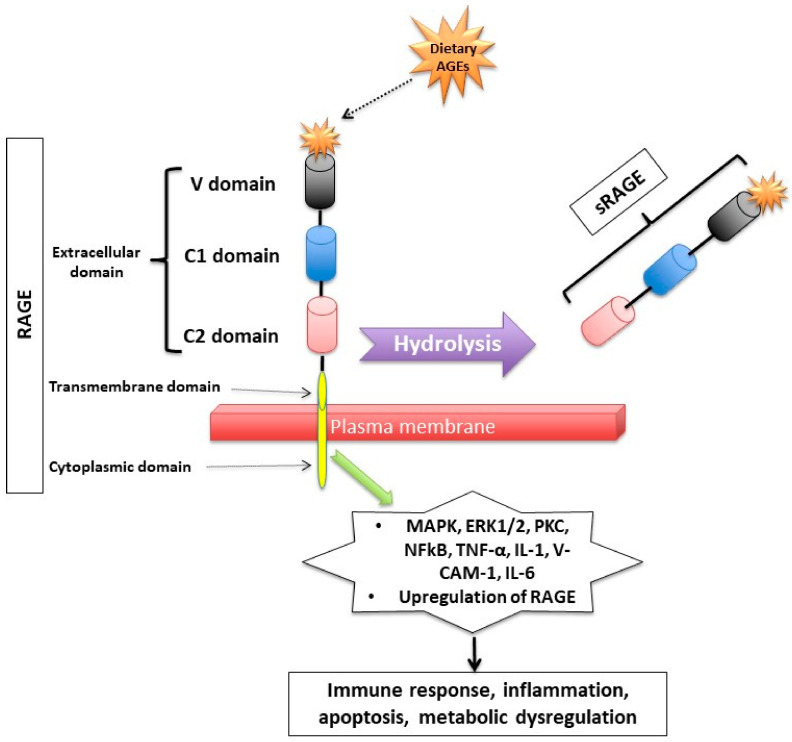
Receptor for Advanced Glycation End (RAGE) products and its mechanism of action. RAGE consists of transmembrane, cytosolic and extracellular domains. The extracellular domain consists of V, C1, and C2 domains. The soluble fragment of RAGE (sRAGE) is produced by hydrolysis of the RAGE receptor and contains the RAGE’s extracellular domain only. The binding of AGEs to RAGE induces a series of inflammatory and apoptotic responses intracellularly and contributes to metabolic dysfunction [32,33,34,44,45,46,47,48,49]. Mitogen-activated protein kinase (MAPK), extracellular signal-regulated kinase1/2 (ERK1/2), protein kinase C (PKC) and nuclear factor kappa B (NF-κB), lysyl oxidase (LOX), tumor necrosis factor (TNF-α), interleukin-1 (IL-1), vascular adhesion molecule-1 (VCAM-1), and interleukin-6 (IL-6).

Dietary AGEs bind to several types of receptors (Figure 2). First, AGEs can act by binding to a receptor called RAGE (*R*eceptor for *A*dvanced *G*lycation *E*nd product) which is member of the immunoglobulin superfamily [31]. The expression of the RAGE protein is detected in human trophoblasts in chorionic villi early in fetal life (such as in endothelial cells of embryonic vessels and alveolar capillaries) and gradually increases after birth and in adulthood [32,33].

RAGE has a triple domain: transmembrane, a cytosolic and an extracellular [34], is expressed in cell membranes of several tissues such as heart, lung, skeletal muscle, the vessel wall and the reproductive system [10,34,35] and can be activated by many other ligands including amyloid β peptide, high-mobility group protein B1 (HMGB1) and the S100 group of protein [36].

After binding to RAGE, there is an activation of several intracellular inflammatory signaling pathways that include mitogen-activated protein kinase (MAPK), extracellular signal-regulated kinase1/2 (ERK1/2), protein kinase C (PKC) and nuclear factor kappa B (NF-κB) [37,38]. The activation of those pathways can lead to inflammatory state, cellular oxidative stress, and cellular damage [31] via upregulating markers of reactive oxygen species (ROS), and inflammatory molecules such as tumor necrosis factor (TNF-α), interleukin-1 (IL-1), vascular adhesion molecule-1 (VCAM-1), and interleukin-6 (IL-6) [39]. Interestingly, the binding of AGEs to RAGE upregulates RAGE expression itself, causing inflammation to get worse [31,40,41,42].

Second, RAGEs have been found to have multiple soluble forms detected mainly in body fluids and blood. The two most common forms are: sRAGE (soluble fragment of RAGE) and esRAGE (endogenous secretory RAGE). The sRAGE is produced by hydrolysis (MMPs and ADAM-10 induced proteolytic cleavage mechanisms [43,44]) of the RAGE receptor at the level of the cell surface and can be detected in the blood and bodily fluids [42,45]. Unlike RAGE, sRAGE contains only the extracellular domain of RAGE [42,45], and unlike RAGE, it has an “anti-inflammatory” action since it holds on to the circulating AGEs, thus inhibiting them from exercising their pro-inflammatory effect by binding to RAGE [46,47,48]. Unlike sRAGE, which is derived from the full-length form of RAGE [43], esRAGE is only derived from a part of the RAGE, specifically from pre-mRNA alternative splicing [49]. The esRAGE, also called variant RAGE-v1, usually comprises 20% of the total soluble RAGE receptors [49].

### 1.3. How Are Dietary AGEs Cleared from the Body?

Dietary AGEs are orally absorbed [50], with approximately 10% of them being absorbed in the GI tract and delivered to the liver and to other organs including but not limited to the reproductive system [51]. Dietary AGEs are mainly cleared by the urinary tract system (kidneys): nearly a third of dietary AGEs are excreted in the urine, with approximately 50% of the AGEs remaining quantified in the urine until approximately a few days following its consumption [52], and accumulating in the body leading to inflammation and oxidative stress [51]. The beginning of AGEs’ degradation occurs mainly intracellularly, therefore they first need to be inserted into the cell. Some of the AGE-receptors that are involved in the detoxification process are the AGE-R1/OST-48, AGE-R3/galectin-3 and scavenger-receptors [53]. These receptors compete with RAGE and try to bind the circulating dietary AGEs, thus they inhibit the toxic RAGE-mediated signaling pathways. The uptake of AGEs takes place through the activation of membrane receptors via phosphorylation or ubiquitinylation of the cytoplasmic side of the receptor, thus inducing its endocytosis [54].

## 2. Polycystic Ovary Syndrome (PCOS) and Dietary AGEs

PCOS is arguably the most common endocrinopathy in reproductive-aged women [55,56]. It is associated with significant metabolic changes and reproductive alterations, making it the most common cause (up to 70%) of anovulation [56]. Most women with PCOS display some type of metabolic dysfunction [57]. Studies have shown that women with PCOS have elevated circulating AGEs, which is exacerbated by exogenous absorption of AGEs from western heat processed diets [58]. AGEs contribute to the pathogenesis of PCOS as well as the consequential metabolic and reproductive system effects as proven by several in vitro experiments, animal models, and human studies [9,42,59,60,61,62].

When quantified at the ovarian tissue level by immunohistochemistry, RAGE and AGE-modified proteins are expressed in women with or without PCOS, though at much different concentrations [9]. There are alterations in the AGE system that have been shown to be related to reproductive impairment in women with PCOS [63]. It was first demonstrated in 2005 that overweight women with PCOS, compared to those without PCOS and independently of the hyperglycemia level (well known to be correlated to an increase in AGEs level), have increased AGEs’ levels and the upregulation of monocyte RAGE expression [42]. Then, in 2008, it was shown that lean women with PCOS without insulin resistance (another factor that is well known to be correlated with elevated body AGEs) also have elevated serum AGE levels compared to women with components of PCOS only (such as hyperandrogenemia with or without PCO-ovarian morphology) [59]. These findings suggest that these harmful molecules and the pro-inflammatory multi-ligand receptor RAGE have a pathological significance in reproductive abnormalities, in particular in ovarian dysfunction, in PCOS. Additionally, several studies in women who underwent IVF, assessed the relationship between sRAGE and PCOS and showed that compared to women without PCOS, those with PCOS had significantly lower sRAGE levels in the follicular fluid [64,65,66,67]. These findings suggest that there are alterations even in the anti-inflammatory sRAGE receptors in women with PCOS.

Other studies have demonstrated that women with PCOS given isocaloric diets high in AGEs for 2 months had significantly higher testosterone, free androgen index, and androstendione levels compared to women with PCOS on two-months low-AGE isocaloric diet [29]. Animal studies in animals confirmed the same findings, where rats put on a high-AGE diet for six months showed elevated AGE deposition in the reproductive system (theca cells), increased RAGE staining in granulosa cells, and higher plasma testosterone levels compared to low-AGE diet rats [16]. In another study, high-AGE diet showed increased plasma testosterone and decreased plasma estradiol and progesterone in female rats compared to their female rats counterparts on low-AGE diets [68]. This underscores an irrefutable correlation between dietary AGEs and hyperandrogenemia, solidifying the hypothesis that lowering dietary AGEs in PCOS could reduce some of the symptomatology of hyperandrogenemia.

At the cellular level, AGEs cause alterations in lysyl oxidase (LOX), which play a significant role in the regulation of ovarian follicular extracellular matrix organization and can explain some of the changes observed in PCOS [69]. In fact, AGE-mediated stimulation of LOX activity leads to the excessive deposition of collagen in the ovaries of women with PCOS [69]. In a rat model, dietary AGEs were able to reduce the activity of protective glyoxalase-I in the ovary of PCOS and that effect that was partially reversed by a diet low in AGEs [70]. These findings suggest that, at the cellular level, it is plausible that AGEs are significantly involved in the pathophysiology of PCOS, partially via LOX and glyoxalase-I [69]. Another study showed that AGEs may alter the enzymatic activity of specific enzymes such as cholesterol side-chain cleavage enzyme cytochrome P450, steroidogenic acute regulatory protein (StAR), 17α-hydroxylase, and 3β-hydroxysteroid dehydrogenase leading to the symptomatology of hyperandrogenism in PCOS [71]. AGEs also exert a direct effect in granulosa cells on the expression and signaling pathways of luteinizing hormone (LH) receptor and anti-Mullerian hormone receptor [71].

## 3. Ovarian Dysfunction

**Figure 3:**
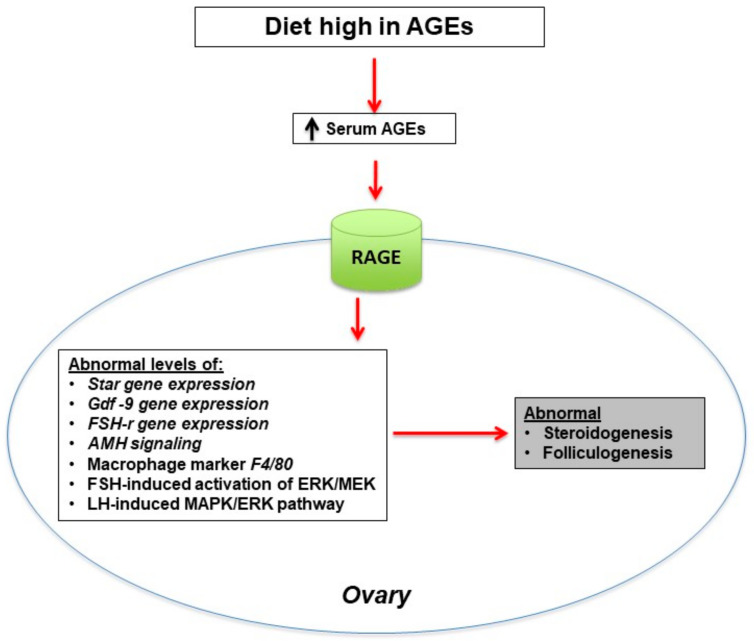
Effect of dietary high levels of advanced glycation end products (AGEs) on ovary function. Diets with large amounts of AGEs increase serum level of AGEs which bind to RAGE in the ovary and cause abnormal expression of genes involved in steroidogenesis and folliculogenesis as well as increase macrophage infiltration in ovarian tissue, ultimately leading to ovarian dysfunction [28,37,72,73,74]. Growth differentiation factor 9 (GDF), follicular stimulating hormone receptor (FSH-r), anti-mullerian hormone (AMH), follicular stimulating hormone (FSH), extracellular-signal-regulated kinase (ERK), mitogen activated protein kinase (MEK), luteinizing hormone (LH), mitogen activated protein kinase (MAPK).

New data indicate that the high-AGE diet could disrupt ovarian function, particularly folliculogenesis and steroidogenesis [18,28,72]. Interestingly, the ingestion of dietary AGEs could also increase ovarian gene expression of inflammatory macrophage markers [72]. A mouse model study evaluated the effect of high-AGE diet on estrous cyclicity and ovarian function. In that study, six-week old C57BL/6J female mice were randomly subjected to either a diet low or high in AGEs for 13 weeks [72] during which daily assessment of estrous cyclicity was performed through vaginal smears, along with oocyte number assessment (following ovarian superovulation with gonadotropins), quantification of genes involved in folliculogenesis, steroidogenesis and ovarian macrophage markers (via whole ovarian tissue mRNA quantification by RT-PCR), and finally ovarian morphology for follicle count. Their results showed that, compared to mice on a diet low in AGEs, mice on a diet high in AGEs spent a significantly longer time in the diestrus phase, had significantly fewer corpora lutea, and showed significant alterations in genes involved in steroidogenesis, in particular an increase in *StAR* mRNA expression levels. They also showed significant alterations in genes involved in folliculogenesis, in particular an increase in growth differentiation factor 9 (*Gdf-9*) and follicular stimulating hormone (FSH) receptor (*FSH-r*) mRNA levels. Mouse macrophage marker *F4/80* mRNA expression was upregulated in mice on a high-AGE diet. These results indicate that folliculogenesis and steroidogenesis could be disrupted by a high-AGE diet, leading to abnormal reproduction in female animals (Figure 3).

At the cellular level, the effect of MAPK/ERK is essential for normal follicle development (regulation of oocyte maturation) and proper ovulation [73]. One study evaluated the in vitro interference of exogenous AGEs with luteinizing hormone (LH)-induced MAPK/ERK signaling pathway in a cell line of KGN cell line granulosa cells [37]. The results of that study showed a direct abnormal effect of exogenous AGEs in this pathway, thus leading to a reduced activation of ERK1/2. Another pathway that was affected by AGEs was the FSH-induced phosphorylation of MEK1/2 and ERK1/2, leading to a reduced activation in the KGN granulosa cells [37]. These data, along with the fact that ERK1/2 activation is crucial for FSH-mediated granulosa cell mitogenesis [74], suggest that exogenous AGEs could potentially alter follicular development (Figure 3).

The effect of AGEs on pathways (such as Smad 1/5/8) and expression of genes (LH receptor [LHR], Anti-Mullerian hormone [AMH] and AMH receptor [AMHR-II]) involved in ovarian follicular development was studied in human luteinized granulosa cells of women who underwent oocyte collection for in vitro fertilization [75]. The AMH signals intracellularly via phosphorylating Smad 1/5/8 [76,77] and plays an important role in normal folliculogenesis by suppressing the differentiation of granulosa cells and follicular development thus protecting them from becoming atretic [78]. In that study, the human luteinized granulosa cells were treated with exogenous AGEs in vitro and showed significant increase in LHR and AMHR-II mRNA levels, but had no change in AMH mRNA expression levels. Compared to luteinized granulosa cells treated without exogenous AGEs in vitro, those treated with exogenous AGEs had significant increase in CYP11A1, 3β-HSD, StAR, and CYP17A1 mRNA expression levels as well as a significant secretion of estradiol [79]. These findings suggest that there is a relationship between exogenous AGEs and steroid expression. KGN granulosa cells treated with recombinant AMH (rAMH) showed a significant increase in the Smad 1/5/8 phosphorylation in the presence of exogenous AGEs in vitro, compared to the absence of AGEs in vitro [80]. Those findings suggest that exogenous AGEs could lead to ovulatory dysfunction partly via the elevated AMH-induced Smad 1/5/8 signaling pathway.

## 4. Perinatal Exposure to Elevated Dietary AGEs and Reproduction in Female Offspring

It is well known that maternal nutrition and the intrauterine environment are crucial in determining susceptibility to reproductive and metabolic disturbances later in life [81,82]. For instance, maternal obesity or consumption of a high fat diet during pregnancy and lactation have been shown to increase the risk of metabolic diseases in offspring [83]. The typical Western diet, commonly consumed by pregnant mothers, contains high amounts of the pro-inflammatory AGEs [27,84,85]. Data have shown that this dietary pattern results in reproductive disturbances [63], oxidative stress and inflammation in both humans and animals [27]. Maternal exposure to high dietary AGEs during pregnancy could predispose mice offspring to metabolic disturbances later in life; for example, perinatal exposure to high dietary AGEs have been shown to predispose the male offspring to weight gain and to metabolic alterations [86]. Additionally, reducing dietary AGEs throughout gestation, lactation, and early postnatal life have been shown to benefit the metabolism (for example pancreatic islet secretion) and the immune system in mice [87].

**Figure 4:**
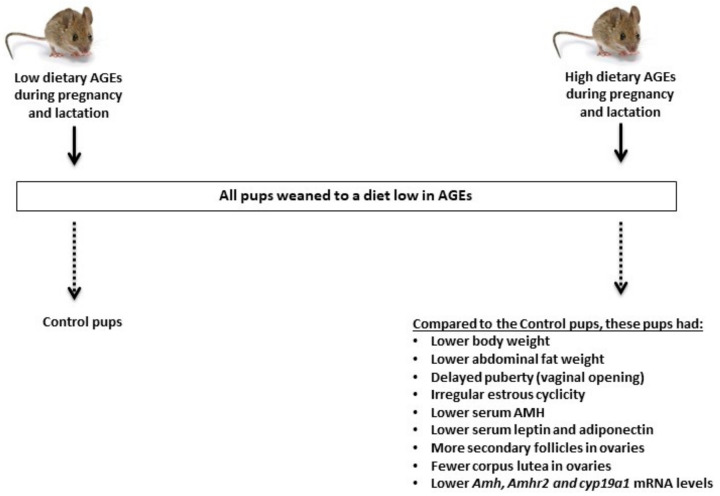
Effect of high dietary AGEs during pregnancy and lactation on reproduction in offspring. Perinatal exposure to high dietary AGEs affects the growth of the offspring as well as the onset of puberty, estrous cyclicity, and ovarian follicular development, including ovulatory events. In addition, perinatal exposure to high dietary AGEs causes the lower expression of ovarian genes involved in folliculogenesis (*Amh* and its receptor *Amhr2*), and steroidogenesis (*Cyp19a1*, which is an aromatase enzyme responsible for conversion of testosterone to estradiol), as well as lower serum AMH levels, which are a marker of ovarian reserve [88]. Antimullerian hormone (AMH), anti-mullerian hormone receptor (anti-mullerian hormone receptor), messenger RNA (mRNA).

In a recent study [88], seven week old female mice were placed on either a diet low or high in AGEs before mating and then during pregnancy and lactation. All offspring were weaned onto a diet low in AGEs and studied until 16 weeks of age by counting them and weighing them at birth and then every week for a total of 11 weeks (Figure 4). The authors assessed the vaginal opening (an indicator of puberty), litter size, growth curve, ovarian follicular count, ovarian gene expression, liver and abdominal fat weights, serum levels of AMH (a marker of ovarian reserve), leptin and adiponectin (markers of adiposity) as well as insulin and glucose tolerance tests in both groups. Their results showed that compared to perinatal exposure to a diet low in AGEs, perinatal exposure to a diet high in AGEs caused lower body weight at birth, delayed growth in adult offspring, lower serum leptin and adiponectin levels, delayed vaginal opening, irregular estrous cyclicity, arrested follicular development, and significant alterations in the expression of genes involved in folliculogenesis such as *Amh* and *Amhr2*, and steroidogenesis such as *Cyp19a1* (aromatase enzyme). These results indicate that perinatal exposure to a diet elevated in AGEs causes deficits in perinatal growth, pubertal onset, and reproductive organ development in female mice.

## 5. Conclusions

With the common ingestion of diets high in AGEs, reproductive-aged women might face more metabolic and reproductive complications. The reproductive function and body energy hemostasis are closely linked at the level of the brain, in particular the hypothalamus, and the ovaries; thus, future studies focusing on the neuroendocrine axis are necessary to further define the mechanisms by which dietary AGEs influence female reproduction. The ingestion of diets high in AGEs causes a systemic state of chronic inflammation, as shown by upregulation of ovarian macrophage marker *F4/80* expression [72], which may directly affect ovarian function. Those dietary AGEs could potentially disrupt the ovarian microenvironment, compromising oocyte competence, formation of healthy embryos, and ultimately conception. Additionally, the elevation of AGEs in the serum and tissues of reproductive-aged women may exacerbate the reproductive dysfunction associated with PCOS.

This review underscores a critical need to unveil, in greater depth, the mechanistic pathways of AGEs, which are molecules present in high amounts in the daily Western diet. Cutting down on the ingestion of AGEs could be demanding and might not be maintainable, thus there is a need to promote therapies targeting AGEs and their intracellular signaling pathways in order to improve ovarian health not only now but in the future generations, since AGEs seem to have transgenerational effects on female reproduction.
